# Supplementary material for: Sex, age, and parental harmonic convergence behavior affect the immune performance of Aedes aegypti offspring
Source: Commun Biol. 2021 Jun 11;4:723. doi: 10.1038/s42003-021-02236-5 (PMC8196008; doi:10.1038/s42003-021-02236-5)
Supplement: Supplementary file 2 — Supplementary Information [file 42003_2021_2236_MOESM2_ESM.pdf]

Supplemental Tables

Table S1: General linear mixed model statistics showing the effects of sex, age, mating status, blood feeding status and parental convergence status as well as their interactions on melanization capability.

	**1d, 3d old unmated mosquitoes**				**3d old mosquitoes**				**5d old females mosquitoes**			
Predictors	*Log-Odds*	*std. Error*	*Statistic*	*p*	*Log-Odds*	*std. Error*	*Statistic*	*p*	*Log-Odds*	*std. Error*	*Statistic*	*p*
not|part	-1.69	0.26	-6.44	**<0.001**	-1.42	0.36	-3.91	**<0.001**	-0.99	0.61	-1.62	0.104
part|full	0.27	0.25	1.05	0.294	0.59	0.36	1.65	0.098	0.71	0.60	1.18	0.239
convergence status	-0.32	0.39	-0.82	0.410	-0.23	0.54	-0.43	0.667	1.12	0.88	1.28	0.202
sex	-1.52	0.23	-6.64	**<0.001**	-1.96	0.41	-4.81	**<0.001**				
age	-0.35	0.21	-1.66	0.098								
convergence status * sex	0.90	0.34	2.66	**0.008**	1.78	0.59	3.00	**0.003**				
convergence status * age	0.31	0.32	0.96	0.337								
sex * age	-0.26	0.31	-0.83	0.405								
convergence status * sex * age	0.60	0.47	1.27	0.202								
mating status					0.04	0.38	0.12	0.906				
convergence status * mating status					0.30	0.57	0.52	0.603				
sex * mating status					0.45	0.54	0.83	0.405				
convergence status * sex * mating status					-0.84	0.80	-1.04	0.297				
blood feeding									0.35	0.44	0.81	0.420
convergence status * blood feeding									0.10	0.69	0.14	0.887
**Random Effects**												
Random intercepts between parental pairs	$0.87_{\text{parental.pair}}$				$1.01_{\text{parental.pair}}$				$2.25_{\text{parental.pair}}$			
Number of parental pairs	$38_{\text{parental.pair}}$				$33_{\text{parental.pair}}$				$18_{\text{parental.pair}}$			
Number of observations	641				409				163			

Table S2: Pairwise comparisons are shown for all possible combinations of sex as main effect on melanisation capability

**comparison**	**Mean Diff.**	**95.00% CI of diff.**	**Adjusted P Value**
female not vs. female part	-0.1880	-0.2989 to -0.07722	**<0.0001**
female not vs. female full	-0.2039	-0.3147 to -0.09311	**<0.0001**
female not vs. male not	-0.2693	-0.3956 to -0.1431	**<0.0001**
female not vs. male part	-0.09485	-0.2211 to 0.03143	0.2642
female not vs. male full	-0.02798	-0.1543 to 0.09829	0.9884
female part vs. female full	-0.01589	-0.1267 to 0.09492	0.9985
female part vs. male not	-0.08129	-0.2076 to 0.04499	0.4400
female part vs. male part	0.09319	-0.03308 to 0.2195	0.2831
female part vs. male full	0.1601	0.03378 to 0.2863	**0.0043**
female full vs. male not	-0.06540	-0.1917 to 0.06088	0.6763
female full vs. male part	0.1091	-0.01719 to 0.2354	0.1347
female full vs. male full	0.1759	0.04967 to 0.3022	**0.0011**
male not vs. male part	0.1745	0.03444 to 0.3145	**0.0053**
male not vs. male full	0.2413	0.1013 to 0.3814	**<0.0001**
male part vs. male full	0.06687	-0.07317 to 0.2069	0.7475

Table S3: General linear mixed model statistics showing the effects of sex, age, mating status, blood feeding status and parental convergence status as well as their interactions on bacterial growth after intra-thoracic injection.

	1d, 3d and 5d old unmated mosquitoes				3d old mosquitoes				5d old, female mosquitoes			
Predictors	*Log-Mean*	*std. Error*	*z-value*	*p*	*Log-Mean*	*std. Error*	*z-value*	*p*	*Log-Mean*	*std. Error*	*z-value*	*p*
(Intercept)	4.61	0.08	57.03	**<0.001**	4.63	0.12	38.13	**<0.001**	3.58	0.18	19.91	**<0.001**
age	0.11	0.04	2.72	**0.006**								
convergence status	-0.41	0.12	-3.47	**0.001**	-0.32	0.17	-1.85	0.065	-0.11	0.25	-0.42	0.674
sex	-1.53	0.08	-18.97	**<0.001**	-1.90	0.15	-12.27	**<0.001**				
age * convergence status	0.29	0.06	5.07	**<0.001**								
age * sex	0.27	0.08	3.63	**<0.001**								
convergence status * sex	0.22	0.12	1.85	0.065								
age* convergence status * sex	-0.28	0.11	-2.53	**0.012**								
mating status					-0.00	0.11	-0.03	0.973				
mating status * sex					0.44	0.21	2.13	**0.033**				
mating status * convergence status					0.29	0.15	1.87	0.061				
sex * convergence status					0.24	0.22	1.09	0.276				
mating status * sex* convergence status					-0.36	0.30	-1.23	0.220				
blood feeding									-1.02	0.23	-4.35	**<0.001**
blood feeding * convergence status									-0.34	0.32	-1.06	0.287
**Random Effects**												
Intercepts between parental pairs	0.48				0.07				0.17			
Marginal R^2 / Conditional R^2	0.55 / 0.584				0.607 / 0.664				0.308 / 0.442			

Number of observations	577	305	136
GLMM family and link	Generalized Poisson (link= “log”)	Generalized Poisson (link= “log”)	Generalized Poisson (link= “log”)

Table S4 Pairwise comparisons are shown for all possible combinations sex, age and convergence status as effects on bacterial growth.

comparison	Mean Diff.	95.00% CI of diff.	Adjusted P Value
1d female converged vs. 3d female converged	-18.98	-54.29 to 16.33	0.8169
1d female converged vs. 5d female converged	-42.91	-82.74 to -3.076	**0.0232**
1d female converged vs. 1d male converged	-20.78	-63.36 to 21.81	0.8942
1d female converged vs. 3d male converged	-80.18	-115.5 to -44.87	**<0.0001**
1d female converged vs. 5d male converged	-85.72	-125.6 to -45.89	**<0.0001**
1d female converged vs. 1d female not converged	-6.674	-49.26 to 35.91	>0.9999
1d female converged vs. 3d female not converged	-22.1	-57.41 to 13.21	0.6303
1d female converged vs. 5d female not converged	-44.45	-83.32 to -5.578	**0.0114**
1d female converged vs. 1d male not converged	-62.41	-105.0 to -19.83	**0.0002**
1d female converged vs. 3d male not converged	-92.01	-127.3 to -56.70	**<0.0001**
1d female converged vs. 5d male not converged	-95.59	-135.4 to -55.76	**<0.0001**
3d female converged vs. 5d female converged	-23.93	-55.87 to 8.007	0.3495
3d female converged vs. 1d male converged	-1.799	-37.11 to 33.51	>0.9999
3d female converged vs. 3d male converged	-61.2	-87.28 to -35.13	**<0.0001**
3d female converged vs. 5d male converged	-66.75	-98.68 to -34.81	**<0.0001**
3d female converged vs. 1d female not converged	12.3	-23.00 to 47.61	0.9906
3d female converged vs. 3d female not converged	-3.122	-29.20 to 22.95	>0.9999
3d female converged vs. 5d female not converged	-25.47	-56.20 to 5.259	0.2085
3d female converged vs. 1d male not converged	-43.44	-78.74 to -8.128	**0.0042**
3d female converged vs. 3d male not converged	-73.03	-99.11 to -46.96	**<0.0001**
3d female converged vs. 5d male not converged	-76.61	-108.5 to -44.67	**<0.0001**
5d female converged vs. 1d male converged	22.13	-17.70 to 61.96	0.783
5d female converged vs. 3d male converged	-37.27	-69.21 to -5.337	**0.0088**
5d female converged vs. 5d male converged	-42.82	-79.69 to -5.938	**0.0094**
5d female converged vs. 1d female not converged	36.23	-3.598 to 76.07	0.1117
5d female converged vs. 3d female not converged	20.81	-11.13 to 52.74	0.5701
5d female converged vs. 5d female not converged	-1.542	-37.38 to 34.30	>0.9999
5d female converged vs. 1d male not converged	-19.51	-59.34 to 20.33	0.8918
5d female converged vs. 3d male not converged	-49.1	-81.04 to -17.17	**<0.0001**
5d female converged vs. 5d male not converged	-52.68	-89.56 to -15.80	**0.0003**
1d male converged vs. 3d male converged	-59.4	-94.71 to -24.10	**<0.0001**
1d male converged vs. 5d male converged	-64.95	-104.8 to -25.11	**<0.0001**
1d male converged vs. 1d female not converged	14.1	-28.48 to 56.69	0.9938

1d male converged vs. 3d female not converged	-1.323	-36.63 to 33.98	>0.9999
1d male converged vs. 5d female not converged	-23.67	-62.55 to 15.20	0.6693
1d male converged vs. 1d male not converged	-41.64	-84.22 to 0.9454	0.0615
1d male converged vs. 3d male not converged	-71.23	-106.5 to -35.93	**<0.0001**
1d male converged vs. 5d male not converged	-74.81	-114.6 to -34.98	**<0.0001**
3d male converged vs. 5d male converged	-5.542	-37.48 to 26.39	>0.9999
3d male converged vs. 1d female not converged	73.51	38.20 to 108.8	**<0.0001**
3d male converged vs. 3d female not converged	58.08	32.00 to 84.16	**<0.0001**
3d male converged vs. 5d female not converged	35.73	5.000 to 66.46	**0.0092**
3d male converged vs. 1d male not converged	17.77	-17.54 to 53.08	0.8729
3d male converged vs. 3d male not converged	-11.83	-37.91 to 14.25	0.9332
3d male converged vs. 5d male not converged	-15.41	-47.34 to 16.53	0.9012
5d male converged vs. 1d female not converged	79.05	39.22 to 118.9	**<0.0001**
5d male converged vs. 3d female not converged	63.62	31.69 to 95.56	**<0.0001**
5d male converged vs. 5d female not converged	41.27	5.435 to 77.11	**0.0104**
5d male converged vs. 1d male not converged	23.31	-16.52 to 63.14	0.7221
5d male converged vs. 3d male not converged	-6.288	-38.22 to 25.65	>0.9999
5d male converged vs. 5d male not converged	-9.866	-46.74 to 27.01	0.9991
1d female not converged vs. 3d female not converged	-15.43	-50.73 to 19.88	0.9481
1d female not converged vs. 5d female not converged	-37.78	-76.65 to 1.096	0.065
1d female not converged vs. 1d male not converged	-55.74	-98.32 to -13.16	**0.0016**
1d female not converged vs. 3d male not converged	-85.34	-120.6 to -50.03	**<0.0001**
1d female not converged vs. 5d male not converged	-88.92	-128.7 to -49.08	**<0.0001**
3d female not converged vs. 5d female not converged	-22.35	-53.08 to 8.382	0.3959
3d female not converged vs. 1d male not converged	-40.31	-75.62 to -5.006	**0.0116**
3d female not converged vs. 3d male not converged	-69.91	-95.99 to -43.83	**<0.0001**
3d female not converged vs. 5d male not converged	-73.49	-105.4 to -41.55	**<0.0001**
5d female not converged vs. 1d male not converged	-17.96	-56.84 to 20.91	0.9246
5d female not converged vs. 3d male not converged	-47.56	-78.29 to -16.83	**<0.0001**
5d female not converged vs. 5d male not converged	-51.14	-86.98 to -15.30	**0.0004**
1d male not converged vs. 3d male not converged	-29.6	-64.91 to 5.710	0.1948
1d male not converged vs. 5d male not converged	-33.18	-73.01 to 6.657	0.2026
3d male not converged vs. 5d male not converged	-3.578	-35.51 to 28.36	>0.9999

Table S5: General linear mixed model statistics showing the effects of sex, age and parental convergence status as well as their interactions on mortality after bacterial challenge.

	**1d, 3d, and 5d old unmated mosquitoes**				**3d old mosquitoes**				**5d old, female mosquitoes**			
Predictors	*Estimates*	*std. error*	*Statistic*	*p*	*Estimates*	*std. error*	*Statistic*	*p*	*Estimates*	*std. error*	*Statistic*	*p*
(Intercept)	-5.72	0.27	-21.40	**<0.001**	-5.57	0.41	-13.56	**<0.001**	-5.86	0.36	-16.29	**<0.001**
age	0.48	0.38	1.26	0.207								
convergence status	0.36	0.28	1.26	0.207								
sex	0.49	0.36	1.35	0.177	0.45	0.56	0.79	0.427	0.63	0.48	1.32	0.185
age * convergence status	0.05	0.27	0.20	0.843								
age * sex	-0.56	0.28	-2.01	**0.044**	-0.71	0.52	-1.36	0.173				
convergence status * sex	-0.08	0.39	-0.21	0.836								
age * convergence status * sex	-0.05	0.20	-0.26	0.799								
mating status					-0.11	0.72	-0.15	0.881				
mating status * sex					-0.37	1.01	-0.37	0.712				
mating status * convergence status					1.00	0.74	1.35	0.177				
sex * convergence status					0.59	0.72	0.82	0.411				
mating status * sex * convergence status					-0.14	0.52	-0.27	0.785				
blood feeding									0.53	0.51	1.04	0.296
blood feeding * convergence status									-0.15	0.69	-0.22	0.823
**Random Effects**												
τ_{00}	0.22 parental.pair				0.22 parental.pair				0.06 parental.pair			
N	16 parental.pair				15 parental.pair				16 parental.pair			
Observations	121				60				29			

Table S6: General linear mixed model statistics showing the effects of parental convergence on dissemination, infectiousness and viral titers after a DENV-2 infectious blood meal.

	**DENV-2 prevalence**			
Predictors	*Log-odds*	*std. Error*	*z-value*	*p*
(Intercept)	0.46	0.11	4.1	**<0.001**
Non-converged (vs converged)	0.10	0.16	0.66	0.510
Viral dissemination to head and legs	-1.82	0.23	-8.00	**<0.001**
Viral infectiousness in saliva	-5.82	1.36	-4.29	**<0.001**
Linear effect of time (i.e., days post-infection)	4.58	1.16	3.97	**<0.001**
Quadratic ("hump-shaped") effect of time	-3.01	1.16	-2.59	**0.010**
Convergence status * viral dissemination to heads and legs	-0.15	0.31	-0.49	0.625
Convergence status * viral infectiousness in saliva	-1.48	3.29	-0.45	0.653
Convergence status * linear effect of time	-2.94	1.62	-1.81	0.065
Convergence status * quadratic effect of time	-3.01	1.16	-2.59	0.097
Viral dissemination to head and legs * linear effect of time	10.74	2.75	3.90	**<0.001**
Viral infectiousness in saliva * linear effect of time	15.35	16.24	0.94	0.345
Viral dissemination to head and legs * quadratic effect of time	-6.55	2.28	-2.87	**0.004**
Viral infectiousness in saliva * quadratic effect of time	-6.47	9.95	-0.65	0.515
Convergence status * viral dissemination to head and legs * linear effect of time	0.47	3.69	0.13	0.898
Convergence status * viral infectiousness in saliva * linear effect of time	20.84	38.06	0.55	0.584
Convergence status * viral dissemination to head and legs * quadratic effect of time	2.19	3.10	0.71	0.479
Convergence status * viral infectiousness in saliva * quadratic effect of time	-5.44	18.91	-0.29	0.774
**Random effects**				
Intercepts between mosquitoes, nested within replicate	0			
Intercepts between replicates	0			
Marginal R^2 / Conditional R^2	0.783 / 0.783			
Number of observations	720			
GLMM family and link	Binomial (link= "logit")			
Log-likelihood (degrees of freedom)	149.08 (20)			

Table S7: Pairwise comparisons are shown for all possible combinations of parental convergence status as main effect on DENV prevalence

comparison	days post infection	sample	estimate (Log-odds)	SE	df	t-ratio	p-value
Converged vs. non-converged	3	head.legs	-0.9294	0.947	88	-0.981	0.3291
Converged vs. non-converged	3	body	-0.8656	0.34	88	-2.547	**0.0126**
Converged vs. non-converged	18	saliva	-0.7939	1.024	88	-0.775	0.4401
Converged vs. non-converged	6	body	-0.2827	0.209	88	-1.353	0.1795
Converged vs. non-converged	18	head.legs	-0.2335	0.346	88	-0.674	0.502
Converged vs. non-converged	15	saliva	-0.2071	0.829	88	-0.25	0.8034
Converged vs. non-converged	18	body	-0.036	0.354	88	-0.102	0.919
Converged vs. non-converged	6	head.legs	-0.0351	0.463	88	-0.076	0.9398
Converged vs. non-converged	9	body	0.0917	0.234	88	0.391	0.6966
Converged vs. non-converged	15	body	0.215	0.217	88	0.992	0.3239
Converged vs. non-converged	12	body	0.2576	0.236	88	1.092	0.2777
Converged vs. non-converged	15	head.legs	0.3825	0.226	88	1.695	0.0935
Converged vs. non-converged	9	head.legs	0.4817	0.266	88	1.81	0.0737
Converged vs. non-converged	12	saliva	0.5905	1.085	88	0.544	0.5878
Converged vs. non-converged	12	head.legs	0.6208	0.258	88	2.404	**0.0183**
Converged vs. non-converged	9	saliva	1.599	2.69	88	0.594	0.5538
Converged vs. non-converged	6	saliva	2.8183	6.022	88	0.468	0.6409
Converged vs. non-converged	3	saliva	4.2485	10.878	88	0.391	0.6971

Table S8: A nested general linear mixed model showing the effects of parental convergence on dissemination and infectiousness over time after a DENV-2 infectious blood meal.

	**DENV-2 prevalence**			
Predictors	*Log-odds*	*std. Error*	*z-value*	*p*
(Intercept)	0.46	0.11	4.10	**<0.001**
Non-converged (vs. converged)	0.10	0.16	0.66	0.510
Converged / Dissemination	-1.82	0.23	-8.00	**<0.001**
Non-converged / Dissemination	-1.97	0.21	-9.41	**<0.001**
Converged / Infectiousness	-5.82	1.36	-4.29	**<0.001**
Non-converged / Infectiousness	-7.30	2.99	-2.44	**0.015**
Converged / Infected / Linear trend over time	4.58	1.16	3.97	**<0.001**
Non-converged / Infected / Linear trend over time	1.64	1.14	1.44	0.151
Converged / Dissemination / Linear trend over time	15.32	2.50	6.13	**<0.001**
Non-converged / Dissemination / Linear trend over time	12.85	2.17	5.92	**<0.001**
Converged / Infectiousness / Linear trend over time	19.94	16.21	1.23	0.219
Non-converged / Infectiousness / Linear trend over time	37.81	34.39	1.10	0.272
Converged / Infected / Quadratic trend over time	-3.01	1.16	-2.59	**0.010**
Non-converged / Infected / Quadratic trend over time	-0.31	1.14	-0.27	0.789
Converged / Dissemination / Quadratic trend over time	-9.55	1.97	-4.86	**<0.001**
Non-converged / Dissemination / Quadratic trend over time	-4.66	1.75	-2.66	**0.008**
Converged / Infectiousness / Quadratic trend over time	-9.49	9.89	-0.96	0.337
Non-converged / Infectiousness / Quadratic trend over time	-12.21	16.03	-0.76	0.446
**Random effects**				
Intercepts between mosquitoes, nested within replicate	0			
Intercepts between replicates	0			
Marginal R^2 / Conditional R^2	0.783 / 0.783			
Number of observations	720			
GLMM family and link	Binomial (link= “logit”)			
Log-likelihood (degrees of freedom)	149.08 (20)			

Supplemental Figures

Figure S1

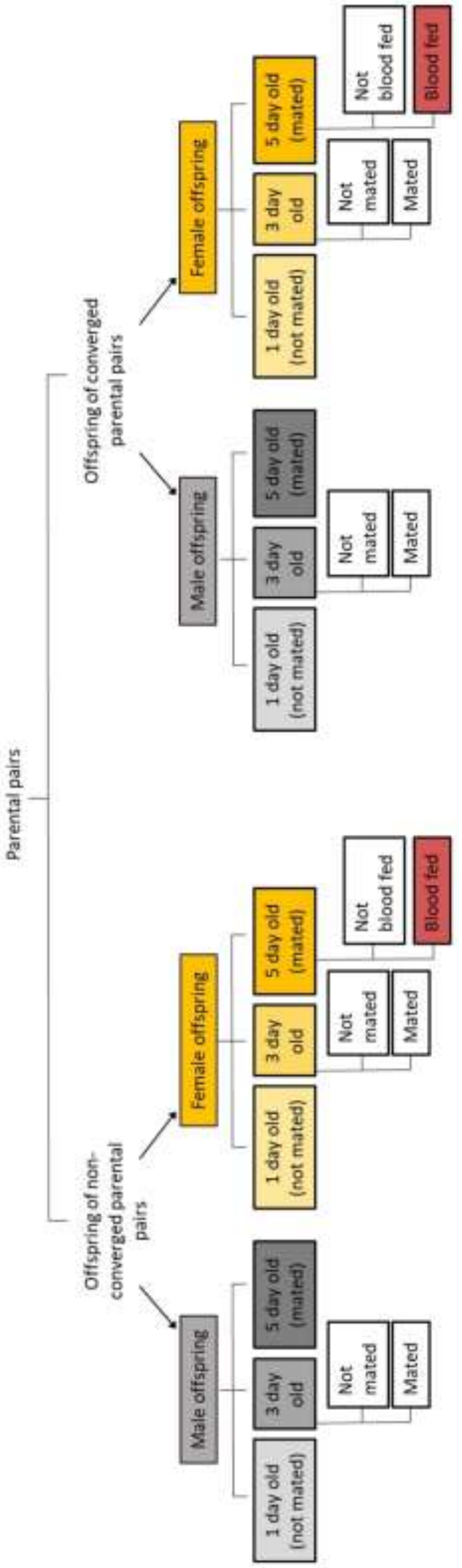

Melanization and bacterial growth assays timeline

dpe	female	male
1	1d injection	1d injection
2	1d sampling	1d sampling
3	3d injection	3d injection
4	3d sampling<br>5d blood feed	3d sampling
5	5d injection	
6	5d sampling	

Fig. S1 Experimental groups for each immune assay. For each group, results from 5 individuals (5 individuals of each gender in mixed-gender groups) per parental pair were obtained. Individuals from 10 converged and 10 not converged parental pairs were investigated.

Fig. S2

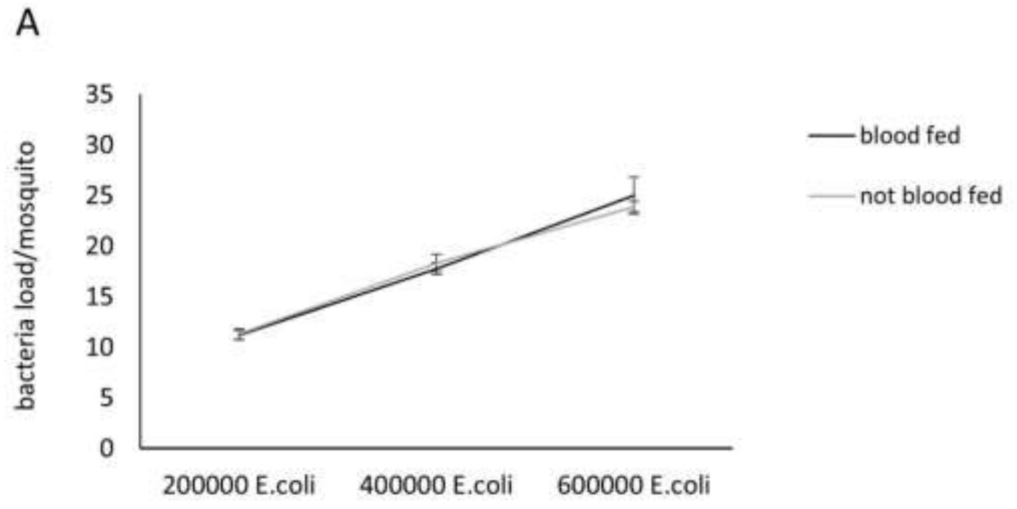

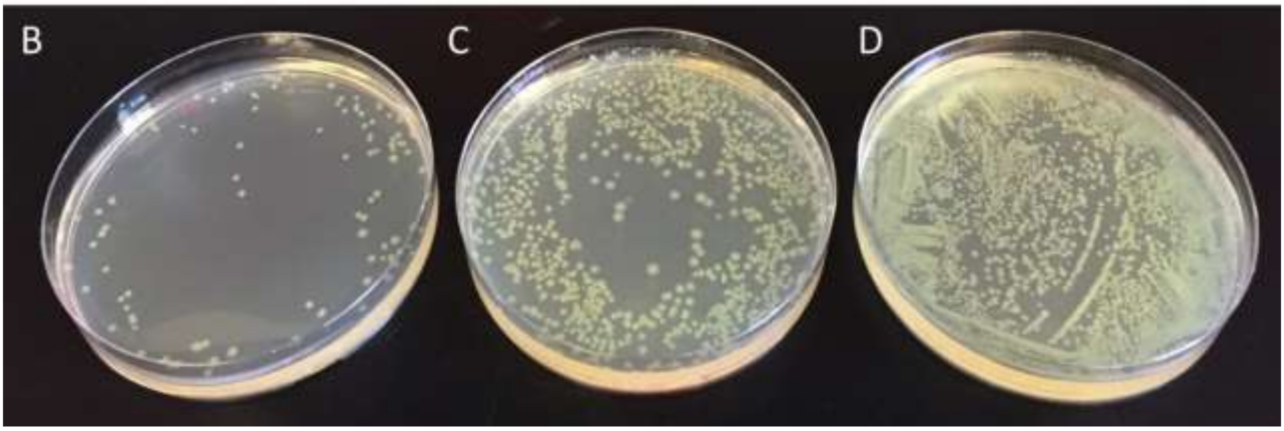

Fig. S2 AU fluorescence values of blood fed and not blood fed female mosquitoes immediately after injection with known doses of GFP expressing *Escherichia coli* (A). Representative LB plates after incubation of samples derived from a blood fed female (B), not blood fed female (C) and male (D) mosquitoes 24h after injection of GFP expressing *E.coli*.

Fig. S3

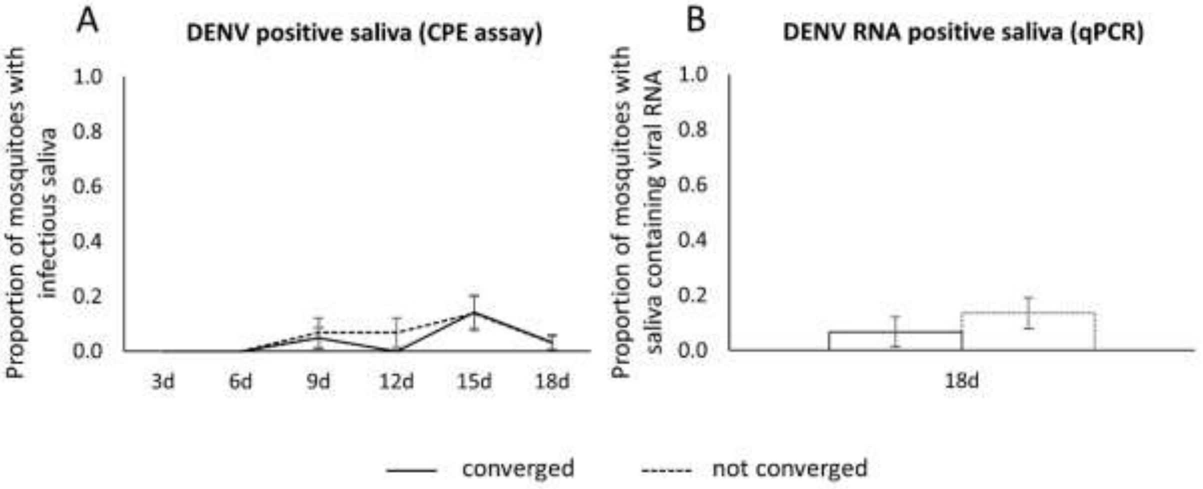

Fig. S3 Presence of dengue-2 virus in mosquito saliva. Cytopathic effect (A) and quantitative PCR (B) data for dengue-2 virus (DENV) screening of saliva samples. Proportion of positive saliva from females from converging (solid line) and not converging (dashed line) parental pairs after ingestion of DENV infectious blood meal on days 3, 6, 9, 12, 15 and 18 post infection. Data points represent mean values with error bars showing standard error of the mean.

Supplemental Methods

Validation of GFP E. coli assay

We validated the method by running three additional experiments:

- Validate comparability of results between different sampling days.

Due to the large sample size and long processing time of the samples it was not possible to inject mosquitoes with bacteria on each sampling day and use those as internal standards on each plate. However, we closely monitored environmental condition during the 5h in-vitro incubation period (time, temperature, shaking speed) and prior to starting the experiments tested how comparable results for this method were between samples and sampling days. We compared those variations to variation between samples processed using a conventional colony forming unit (CFU) assay and found no difference. In short, samples were plated on LB agar plates and plates were incubated over night at 37○C, CFUs were counted.

- Validate GFE fluorescence readings by comparing to results from a CFU assay.

To exclude that composition of the mosquitoes themselves (male, female, blood fed females) might differently interfere with bacterial growth during the 5h incubation period in LB medium, blood fed females, females and males were injected with 200,000 E.coli bacteria and homogenized as described after 24h. The number of CFU correlated with AU fluorescence readings obtained from GFP measurements between those three groups (Figure S2).

- Validate that components of an undigested blood meal do not interfere with in-vitro bacterial growth during the 5h incubation period.

To account for any interference from the partially digested blood meal with the fluorometric measurement of bacteria concentration, blood fed and not blood fed animals were injected on day 2 after a blood meal with three different bacteria concentrations and immediately immobilized on ice and homogenized. The homogenate was subsequently incubated for 5h in LB media. Samples were measured and values for bacterial load in blood fed and un-fed females were compared. No difference in the fluorescent signal intensity between samples deriving from blood fed and un-fed females was found if females were homogenized immediately after inoculation. We can therefore assume that any difference in bacterial growth between fed and un-fed females originates from physiological difference between those experimental groups and not from an experimental artefact.
