# Supplementary material for: Sex, age, and parental harmonic convergence behavior affect the immune performance of Aedes aegypti offspring
Source: Commun Biol. 2021 Jun 11;4:723. doi: 10.1038/s42003-021-02236-5 (PMC8196008; doi:10.1038/s42003-021-02236-5)
Supplement: Supplementary file 3 — Reporting summary [file 42003_2021_2236_MOESM3_ESM.pdf]

nature research

Corresponding author(s): Courtney Murdock

Last updated by author(s): Apr 16, 2021

Reporting Summary

Nature Research wishes to improve the reproducibility of the work that we publish. This form provides structure for consistency and transparency in reporting. For further information on Nature Research policies, see our Editorial Policies and the Editorial Policy Checklist.

Statistics

For all statistical analyses, confirm that the following items are present in the figure legend, table legend, main text, or Methods section.

n/a	Confirmed	
☐	☒	The exact sample size (n) for each experimental group/condition, given as a discrete number and unit of measurement
☐	☒	A statement on whether measurements were taken from distinct samples or whether the same sample was measured repeatedly
☐	☒	The statistical test(s) used AND whether they are one- or two-sided<br>*Only common tests should be described solely by name; describe more complex techniques in the Methods section.*
☐	☒	A description of all covariates tested
☐	☒	A description of any assumptions or corrections, such as tests of normality and adjustment for multiple comparisons
☐	☒	A full description of the statistical parameters including central tendency (e.g. means) or other basic estimates (e.g. regression coefficient) AND variation (e.g. standard deviation) or associated estimates of uncertainty (e.g. confidence intervals)
☐	☒	For null hypothesis testing, the test statistic (e.g. F, t, r) with confidence intervals, effect sizes, degrees of freedom and P value noted<br>*Give P values as exact values whenever suitable.*
☒	☐	For Bayesian analysis, information on the choice of priors and Markov chain Monte Carlo settings
☐	☒	For hierarchical and complex designs, identification of the appropriate level for tests and full reporting of outcomes
☒	☐	Estimates of effect sizes (e.g. Cohen's d, Pearson's r), indicating how they were calculated

Our web collection on statistics for biologists contains articles on many of the points above.

Software and code

Policy information about availability of computer code

Data collection	Raven 1.0 software, Cornell Laboratory of Ornithology, Ithaca, NY
Data analysis	RStudio: Integrated Development Environment for R. Boston, MA: RStudio, Inc.; 2016

For manuscripts utilizing custom algorithms or software that are central to the research but not yet described in published literature, software must be made available to editors and reviewers. We strongly encourage code deposition in a community repository (e.g. GitHub). See the Nature Research guidelines for submitting code & software for further information.

Data

Policy information about availability of data

All manuscripts must include a data availability statement. This statement should provide the following information, where applicable:

- Accession codes, unique identifiers, or web links for publicly available datasets
- A list of figures that have associated raw data
- A description of any restrictions on data availability

The data that support the findings of this study are available from the corresponding author upon reasonable request and have been deposited within Dryad (https://doi.org/10.5061/dryad.ffbg79ct0)
Figures with associated raw data: Figure 1, 2 and 3 in main text; figures S2 and S3 in SI material

Field-specific reporting

Please select the one below that is the best fit for your research. If you are not sure, read the appropriate sections before making your selection.

☐ Life sciences ☐ Behavioural & social sciences ☒ Ecological, evolutionary & environmental sciences

For a reference copy of the document with all sections, see nature.com/documents/nr-reporting-summary-flat.pdf

Ecological, evolutionary & environmental sciences study design

All studies must disclose on these points even when the disclosure is negative.

Study description	In this study, we explore the relationship between harmonic convergence (a mechanism used by females to assess and select among potential mates) and offspring immune function in the yellow fever mosquito, Aedes aegypti. We utilize a variety of immune challenges, as well as dengue-2 virus infection, to explore the ramifications of harmonic convergence on evolutionarily conserved humoral and cellular immune responses and metrics of vector competence.
Research sample	The study was carried out on Aedes aegypti - an important vector of several emerging pathogenic arboviruses including dengue, chikungunya, and Zika viruses. We investigated the effect of parental harmonic convergence status off offspring of different age (1d, 3d, 5d), sex (male vs female), mating status (mated vs unmated) and blood feeding status (blood fed vs not blood feed).
Sampling strategy	Power calculations were performed using similar experimental setup as metric to determine expected effect sizes.
Data collection	All data were collected by the first author on the manuscript. Data collection was performed using observational data collection (melanization assay), instrument based data collection (Varioskan, Thermo Scientific, Waltham, MA, USA, bacterial growth) and cell culture based assays (plaque assays, DENV infections).
Timing and spatial scale	Data collection began in December 2016 and ended July 2018. Assays were performed sub sequentially (1. melanization assay, 2. bacterial growth, 3. dengue-2 virus infections).
Data exclusions	No data were excluded
Reproducibility	All assays were performed in biological replicates and analyzed using technical replicates
Randomization	Mosquitoes were allocated into treatment groups (converged vs non-converged) based on parental mating behavior. Allocation for biological repeats was random.
Blinding	Blinding was not possible as all experiments were carried out by only one person.

Did the study involve field work? ☐ Yes ☒ No

Reporting for specific materials, systems and methods

We require information from authors about some types of materials, experimental systems and methods used in many studies. Here, indicate whether each material, system or method listed is relevant to your study. If you are not sure if a list item applies to your research, read the appropriate section before selecting a response.

Materials & experimental systems

n/a	Involved in the study	
☒	☐	Antibodies
☐	☒	Eukaryotic cell lines
☒	☐	Palaeontology and archaeology
☐	☒	Animals and other organisms
☒	☐	Human research participants
☒	☐	Clinical data
☒	☐	Dual use research of concern

Methods

n/a	Involved in the study	
☒	☐	ChIP-seq
☒	☐	Flow cytometry
☒	☐	MRI-based neuroimaging

Eukaryotic cell lines

Policy information about cell lines

Cell line source(s)	Vero cell line
Authentication	*Describe the authentication procedures for each cell line used OR declare that none of the cell lines used were authenticated.*
Mycoplasma contamination	All cell lines tested negative for mycoplasma
Commonly misidentified lines (See ICLAC register)	*Name any commonly misidentified cell lines used in the study and provide a rationale for their use.*

Animals and other organisms

Policy information about studies involving animals; ARRIVE guidelines recommended for reporting animal research

Laboratory animals	Aedes aegypti, originating from Kamphaeng Phet Province, Thailand
Wild animals	study did not involve wild animals
Field-collected samples	study did not involve animals collected in the field
Ethics oversight	no ethical approval required

Note that full information on the approval of the study protocol must also be provided in the manuscript.
